# Modulated Gut Microbiota for Potential COVID-19 Prevention and Treatment

**DOI:** 10.3389/fmed.2022.811176

**Published:** 2022-03-03

**Authors:** Shuai Zhao, Pengya Feng, Wenbo Meng, Weilin Jin, Xun Li, Xiangkai Li

**Affiliations:** ^1^Intersection Laboratory of Life Medicine, School of Life Sciences, Lanzhou University, Lanzhou, China; ^2^Medical Frontier Innovation Research Center, Institute of Cancer Neuroscience, The First Hospital of Lanzhou University, The First Clinical Medical College of Lanzhou University, Lanzhou, China

**Keywords:** SARS-CoV-2, COVID-19, gut microbiota, immune system, probiotic

## Abstract

COVID-19, caused by severe acute respiratory syndrome coronavirus 2 (SARS-CoV-2) has gained global attention. SARS-CoV-2 identifies and invades human cells *via* angiotensin-converting enzyme 2 receptors, which is highly expressed both in lung tissues and intestinal epithelial cells. The existence of the gut-lung axis in disease could be profoundly important for both disease etiology and treatment. Furthermore, several studies reported that infected patients suffer from gastrointestinal symptoms. The gut microbiota has a noteworthy effect on the intestinal barrier and affects many aspects of human health, including immunity, metabolism, and the prevention of several diseases. This review highlights the function of the gut microbiota in the host's immune response, providing a novel potential strategy through the use of probiotics, gut microbiota metabolites, and dietary products to enhance the gut microbiota as a target for COVID-19 prevention and treatment.

## Introduction

Emerging respiratory infectious diseases are among the top concerns and fascinations of both the public and scientific/medical communities (1). Since December 2019, coronavirus disease 2019 (COVID-19) has become an international public health emergency caused by severe acute respiratory syndrome coronavirus 2 (SARS-CoV-2). The clinical symptoms of the diseases are fever, cough, and myalgia/fatigue, which can develop into acute respiratory distress syndrome (ARDS), and resulting in death (2). Gastrointestinal symptoms, such as diarrhea, have also been reported in several infectious patients (3).

Respiratory viral infections, such as influenza, are often accompanied by intestinal symptoms, mainly including shifts in the intestinal microbiota composition (4, 5). The gut microbiota provides the human host with various biological functions, including metabolizing nutrients, maintaining the normal function of the intestinal mucosal barrier, and promoting immune system development (6). For instance, the gut microbiota can generate anti-inflammatory metabolites including short-chain fatty acids (SCFAs), tryptophan, and niacin (7), promoting CD4 immune cells/Th17 cell proliferation and differentiation (8); and enhance the host's antiviral immune response (9). Thus, balance gut microbiota is associated to maintain systemic health that contributes to resistance to SARS-CoV-2 invasion. This review focuses on the etiology and clinical features, especially intestinal symptoms, of COVID-19 and discusses their effects on the intestinal microecology and the applicable mechanisms whereby the gut microbiota regulates immune and inflammatory responses to coronavirus-related diseases of the host.

## Symptoms of Viral Infections in COVID-19

**Figure 1:**
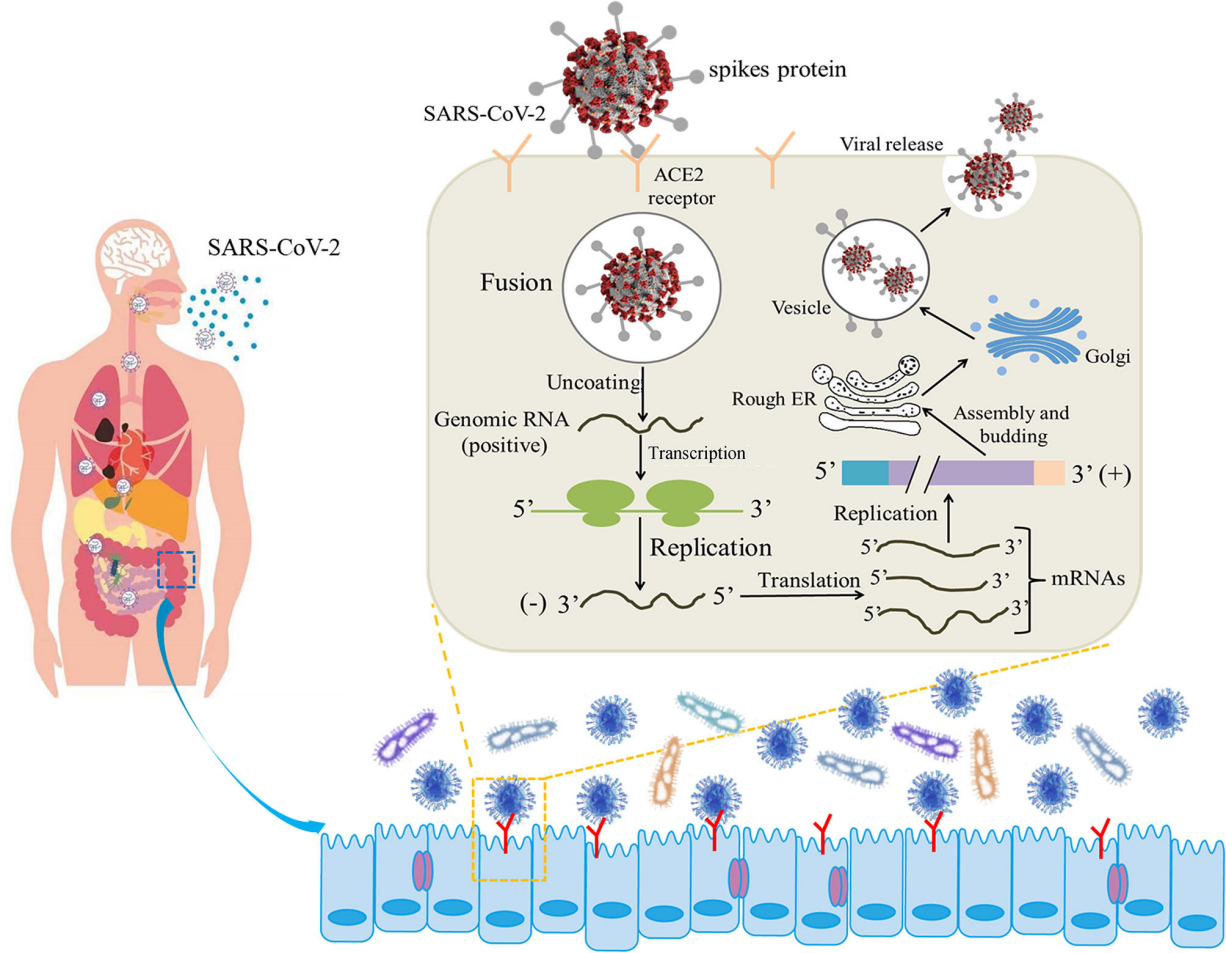
Illustrative model of the entire mechanism of pathogenicity of SARS-CoV-2 (the life cycle of the virus in host cells from attachment to replication) (12, 13).

COVID-19 infection shows non-specific incipient symptoms, and the most common clinical manifestations are fever and dry cough (10). Furthermore, gastrointestinal symptoms, such as diarrhea, have been reported as common manifestations in COVID-19-infected patients (11). Coronavirus entry into host cells is mediated by the transmembrane S-glycoprotein that forms homotrimers protruding from the viral surface (12). The S-glycoprotein S^B^ of SARS-CoV-2 recognizes the angiotensin-converting enzyme 2 (ACE2) receptor on the cell surface, fuses with the cell membrane, and enters the target cell for subsequent replication and assembly (13) (Figure 1). ACE2 is expressed not only in lung tissue but also in esophageal and intestinal epithelia (14). ACE2 is a relevant player in the renin-angiotensin system (RAS), counterbalancing the deleterious effects of angiotensin II. Furthermore, intestinal ACE2 functions as a chaperone for the aminoacid transporter B0AT1. It has been suggested that B0AT1/ACE2 complex in the intestinal epithelium regulates gut microbiota (GM) composition and function, with important repercussions on local and systemic immune responses against pathogenic agents, namely viruses. A previous study pointing that ACE2 imbalance is a key player for the poor outcomes in COVID-19 patients with age-related comorbidities (15).

Additionally, the gut microbiota dysbiosis of COVID-19 is associated with infection severity, and patients suffering from diarrhea increasingly need respiratory assistance and intensive care (16). Thus, diarrhea should be considered a symptom of a possible coronavirus infection and examined for an early diagnosis. Patients showing digestive symptoms receive an average of 9-day treatment from symptoms until admission, whereas patients showing respiratory symptoms receive an average of 7.3-day treatment, indicating that patients showing digestive symptoms spend a longer time in the hospital (17). Furthermore, it is widely known that the gut microbiota is usually influenced by diet, age, and even gender, which means the immunity of a host is also the difference. A previous study showed that gut microbiota diversity is decreased in old age and in patients with certain chronic diseases, which constitute two of the primary fatality groups in COVID-19 infections, it can be assumed that the gut microbiota may play a role in COVID-19 pathology and fatality rate (18).

As for intestinal barrier integrity, inborn and adaptive immune cells can activated and secrete proinflammatory cytokines to the circulatory system, resulting in systemic inflammation (19). COVID-19 patients who suffer from diarrhea and have increased serum interleukin (IL)-6 levels show higher calprotectin concentrations (20). Calprotectin a clinical biomarker for inflammatory bowel diseases and has immunoregulatory functions that could play a potential role in monitoring the disease in the diagnosis and especially the follow-up of COVID-19-related diarrhea (21). In addition, diarrhea may rank second to virus-induced inflammation due to the access of inflammatory cells to the intestinal mucosa, such as neutrophils and lymphocytes, thereby disrupting the gut microbiota (22).

## Gut Microbiota Changes in COVID-19 Patients

Respiratory viral infections often occur with intestinal symptoms. The composition of intestinal microbiota changes in different lung diseases has been reported (23–25). Several studies have reported gut microbiota changes in the feces of patients suffering from COVID-19 (26, 27). Alterations in the gut microbiota were first reported using shotgun metagenomic sequencing that analyzed fecal samples from admission to discharge (27). Cases were classified as mild (no radiographic evidence of pneumonia), moderate (presence of pneumonia), serious (respiratory rate ≥ 30/min or oxygen saturation ≤ 93% while breathing ambient air), or crucial (respiratory failure needing mechanical ventilation, shock, or organ failure needing intensive care). Microbiome results were compared to cases of community-acquired pneumonia and healthy people, characterized by an enrichment of opportunistic pathogens and depletion of beneficial commensals, at the time of hospitalization (27). Another study compared COVID-19 patients, H1N1 patients, and healthy controls to recognize variations in the gut microbiota (26). The same results were also observed in the studies, with significant differences in fecal microbiomes compared to the control groups with characteristics of enriched opportunistic pathogens and depleted beneficial commensals.

**Figure 2:**
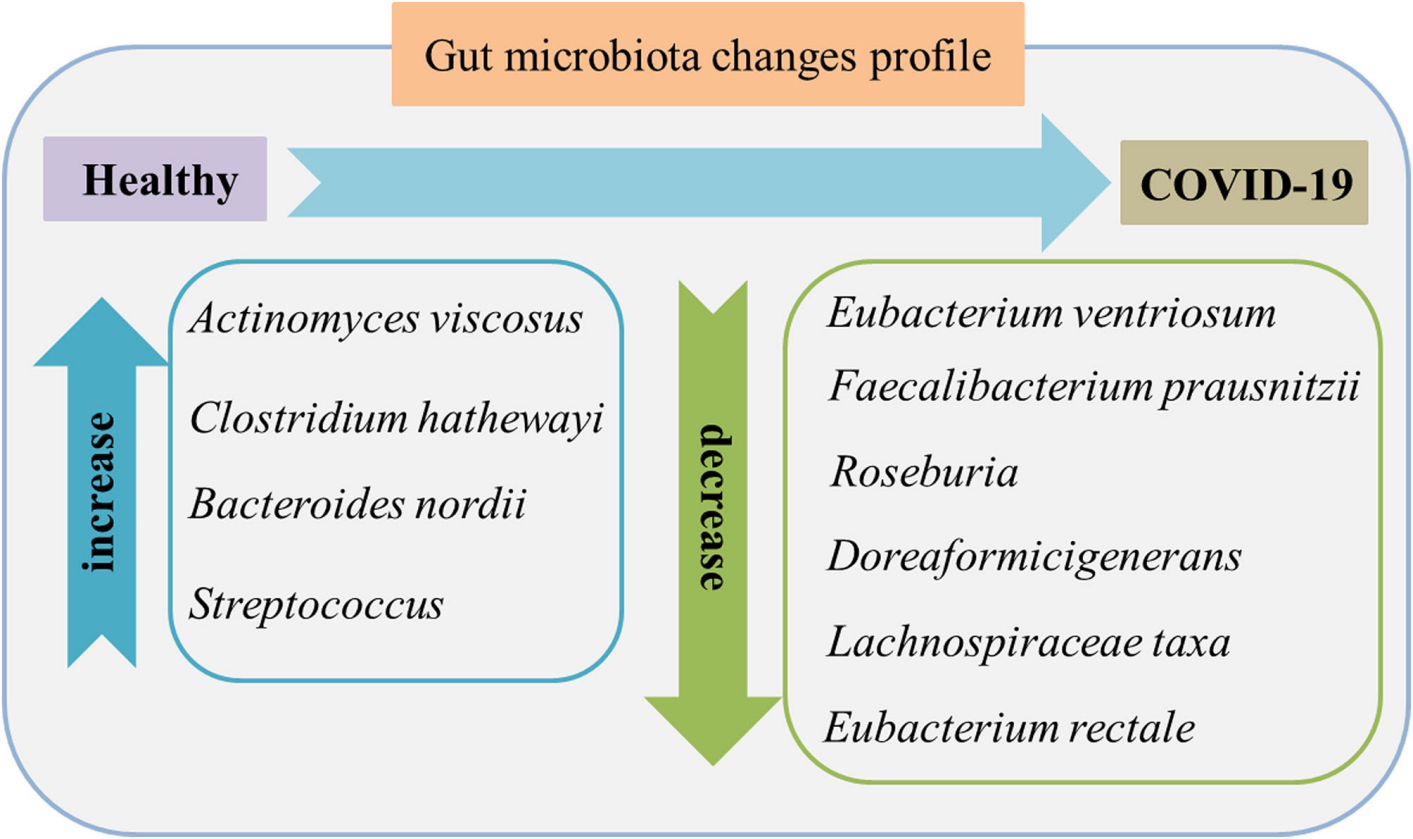
Gut microbiome features in the feces of patients with COVID-19 compared to healthy individuals. The characteristics are enriched opportunistic pathogens and depleted beneficial commensals. Data were collected from published literature (26–28).

Figure 2 shows the main gut microbiota changes after infection. In which the gut microbiota composition shows stratification with disease severity in line with increased concentrations of inflammatory cytokines and blood markers, including C-reactive protein, lactate dehydrogenase, aspartate aminotransferase, and γ-glutamyl transferase (28). There is an association between the relative abundance of *Coprobacillus, Clostridium ramosum*, and *Clostridium hathewayi* and the severity of COVID-19 but an inverse relationship between abundant *Faecalibacterium prausnitzii* (which possess anti-inflammatory effects) and the disease severity. *F. prausnitzii* is a commensal bacterium identified as anti-inflammatory based on human clinical data (29). *F. prausnitzii* has anti-inflammatory effects both *in vitro* and *vivo*. As one of its mechanisms of inhibition and inflammation*, F. prausnitzii* secretes bioactive molecules that can block the activation of nuclear factor-κB and the production of IL-8 by intestinal epithelial cells (30). In addition, *F. prausnitzii* can stimulate high secretion levels of IL-10 through peripheral blood mononuclear cells, mucosal dendritic cells (DCs), and macrophages. It probably plays a role in intestinal homeostasis by inhibiting the production of proinflammatory cytokines, including interferon-γ, tumor necrosis factor-α, IL-6, and IL-12, and enhancing the suppressive activity of forkhead box P3 regulatory T cells (Tregs) in the mucosa (31). *F. prausnitzii* is a known strain with immunomodulatory potential and can contribute to host defense, which was significantly depleted in patients with COVID-19 infected when compared with non-COVID-19 (32). During hospitalization, several particular intestinal microorganisms, *Bacteroides thetaiotaomicron, Bacteroides dorei*, and *Bacteroides massiliensis* downregulating ACE2 expression in the patient's gut, showed a reverse tendency compared to the load of SARS-CoV-2 among fecal samples of the patients. Notably, *Coprobacillus* strongly upregulated the colonic expression of ACE2 in the murine gut in the previous study (33).

Collectively, these findings suggested that the configuration of the gut microbiome may influence the susceptibility and response to SARS-CoV-2 infection and highlighted a known concept that an original and specific method of modulating the gut microbiota may stand for a therapeutic avenue for COVID-19 and its comorbidities. The persistence of depleted symbionts and gut dysbiosis differed from individuals who considered healthy after clearing SARS-CoV-2, as decided by throat swabs and the resolution of respiratory symptoms. In a recent study, 55 of the 406 cases were tested by anal swab, and results showed that 81.8% of the stool samples were positive. It is noteworthy that 23.3% of the stools were still positive after the respiratory viral nucleic acid tested negative, suggesting that the digestive tract clears viruses slower than the respiratory tract (34–36). In the follow-up of patients who recovered from SARS-CoV-2, it has been reported that common clinical sequelae, including general symptoms (49.6%), respiratory symptoms (39%), cardiovascular-related symptoms (13%), psychosocial symptoms (22.7%), and alopecia (28.6%) (37). Furthermore, some patients retained intestinal microecology dysbiosis after recovery, suggesting that the gut microbiota is closely correlated with the host's immune system during infection and after recovery (28). Therefore, daily intake of beneficial substances, such as probiotics, can improve the gut microbiota composition and enhance the body's immunity, thereby contributing to the prevention and treatment of coronavirus and the alleviation of sequelae (38).

## Intestinal Barrier Regulated by the Gut Microbiota for COVID-19 Prevention

The interface between a person and their external environment in the body, the intestinal tract has two key functions. A filter with selected permeability, permits the transfer of required nutrients from the intestinal lumen into circulation and the internal milieu in general. The intestinal tract as a barrier stops the penetration and prevents the movement of some harmful particles, including microorganisms, viruses, and luminal proinflammatory elements (39).

Barrier defenses comprise both immunogenic and non-immunogenic mechanisms, in which the gut microbiota is the main functional component. Regarding non-immunogenic mechanisms, the primary function is selective intestinal permeability influenced by variations in the gut microbiota. Several commensal bacteria, including *Escherichia coli, Klebsiella pneumoniae*, and *Streptococcus viridans*, significantly increase intestinal permeability, allowing harmful entities, such as viruses, to enter circulation more easily, whereas probiotics, such as *Lactobacillus brevis*, have the counteractive effect, decreasing permeability (40). The intestinal tract is also an active immunological organ with more resident immune cells than anywhere else in the body; the gut microbiota contributes to non-immunological defenses in the mucosa (41). The homeostasis of the gut microbiota is beneficial to maintain the cooperation between the innate and adaptive immune systems of the host (42). The innate and adaptive immune systems participate in SARS-CoV-2 infections. Lymphopenia with greatly decreased B and T cells and programmed cell death-1 is upregulated in severely infected patients (43). Various interventions displayed that the gut microbiota increases antiviral immunity (Table 1). It is supposed that the gut microbiota significantly regulates the growth and function of the innate and adaptive immune systems by influencing immune cells to activate anti-inflammatory responses and keep immune homeostasis, influencing the host's susceptibility to different diseases (52, 53). The proinflammatory response should also be considered because the balance between proinflammatory and anti-inflammatory responses is important in many diseases, and understanding how the microbiota shapes immune responses is critical for human health (54). A previous study reported that microbial colonization might provide pro-inflammatory signals that affect the reciprocal development of T-helper and Treg cells. It is suggested that the normal gut microbiota can activate T-cell responses and activate antiviral responses in macrophages by activating inflammasomes and inducing the migration of DCs (9). These findings revealed that the intestinal microbiota acts as a barrier that profoundly impacts the balance between pro-inflammatory and anti-inflammatory immune responses.

**Table 1:** Antiviral functions of the gut microbiota.

**Bacterial species**	**Intervention**	**Mechanisms**	**References**
Commensal microbiota	Antibiotic treatment	Regulates the generation of virus-specific CD4 and CD8 T cells Provides signals leading to the expression of mRNA for pro–IL-1β and pro–IL-18	(44)
Commensal microbiota	Antibiotic treatment	Enhances primary alveolar macrophage function	(45)
Commensal microbiota	*Streptococcus pneumoniae* treatment	Modifies neutrophil phenotype through down-regulating neutrophil expression of an efferocytosis-inhibitory molecule reduces susceptibility to severe pneumonia	(46)
*Bacteroides* species increased	SCFA treatment	Enhancement of CD8^+^T cell metabolism Increased generation of macrophages with reduced ability to produce CXCL1 in airways Reduced neutrophil recruitment, resulting in the attenuation of lung immunopathology	(47)
*Clostridium orbiscindens*	Antibiotic treatment	Enhanced type I IFN signaling in macrophages	(48)
Commensal microbiota	Microbiota transfer	Production of virus-specific CD8^+^T cell responses via dendritic cells	(49)
*Lachnospiraceae* spp.	SCFA treatment	GPR43-mediated and IFNAR dependent IFN-β responses in lung epithelial cells	(50)
*Lactobacillus plantarum* L-137	SCFA treatment	Proinflammatory activity Th1 immune response	(51)

The gut microbiota and its metabolites provides protect against thousands of pathogens and closely associates with the host's systemic and pulmonary immune functions (55). SARS-CoV-2 is a reported respiratory pathogen, but intestinal symptoms should also be considered because the intestinal system associates with the immune system. The intestinal and respiratory microbiota develops simultaneously after birth, and the lungs are inhabited by a microbial population distinct from the gut (56). The interaction between the host and the microbiota shapes different local cell functionalities, immune responses and can further influence disease development (57). Pulmonary immune homeostasis is maintained by a network of tissue-resident cells while ensuring that efficient and rapid immune responses can be mounted against invading pathogens (58).

The function of pulmonary immune homeostasis mainly depends on the respiratory mucosa regulated by the microbiota and systemic metabolome (59). Furthermore, a previous study demonstrated a vital cross-talk between the lungs and intestinal mucosal sites of the human body through what is commonly referred to as the “gut-lung axis” (5). The human oral microbe also plays vital roles in the development and maintenance of immune homeostasis (60). Accumulated evidence for the oral-gut axis has revealed its role in modulating the pathogenesis process in numerous diseases (61). It is compelling to look into the oral and intestinal microbiome combined with SARS-CoV-2 infection and the crosstalk among them, which may provide an improved understanding of the initiation of viral infection and the path of disease deterioration. The gut microbiota has been shown to influence pulmonary immunity (62). An important player in this gut-immune-lung axis is the microbiota that utilizes dietary components as energy sources, and the resulting metabolic byproducts can be potent immune modulators (63). The movement of immune cells between the gut and lungs can be made through the lymphatic system and/or the blood, thereby modulating the immune response of both organs (64). A number of studies indicated that alterations of early-life microbiota, especially gut microbiota, have consequences for lung diseases. A previous study reported that treatment of mice with vancomycin led to a drop in the diversity of the gut microbiota, which was linked to an exaggerated Th2-driven allergic airway inflammation (65). In recent years, studies have pointed out that it is difficult to distinguish the effects of the intestines, followed by the lungs and the host immune system; in all likelihood, both organs will be significantly important (58). Separating early-life events that influence the gut and the immune system and those that directly influence the lungs is difficult. This relationship is bidirectional, and gut microbiology and physiology could be changed by chronic and acute lung diseases (66). Therefore, in the case of a disrupted intestinal mucosal immune block, invading organisms can access the blood or lungs, leading to ARDS (67). Together, these findings showed that the intestinal tract closely associates with the body's antiviral function, showing promise as a target to resist coronavirus infections.

## Probiotics Regulated Gut Microbiota for COVID-19 Treatment

The probiotics function has gained more attention to mediate the gut microbiota exerts an effect on the treatment of environmental contaminants and diseases (68). Probiotics can alter the gut microbiota composition and play essential roles in maintaining the gut microbiota ecosystem (69). Furthermore, several probiotics drive anti-inflammatory and immunoregulatory roles in the settings of enteric infections and mucosal inflammation (70). Although the immune responses caused by bacteria are different from the virus, several previous studies reported that probiotic or gut microbiota is contributed against COVID-19. Probiotics balancing the local microbiota by inhibiting the growth of pathogenic microorganisms (71), and by enhancing local and systemic immune responses (72). They may also influence the composition and activity of microbiota in the intestinal contents. Considering the beneficial effects of probiotics in virus infections, specific probiotics have been suggested to be effective in alleviating the duration and severity of acute rotavirus gastroenteritis (73). A previous study demonstrated that the genus *Lactobacillus* can drive respiratory immune responses to increase the host's defense against respiratory infections (74). Recently, large-scale vaccinations have been implemented worldwide to control COVID-19; however, the discovery of mutant viruses may affect vaccine immunity. Furthermore, some studies reported that the gut microbiota could act as immune modulators and natural vaccine adjuvants to affect intestinal immune responses (75). Intestinal dysbiosis play a role in the failure to respond to vaccines (75). In the management of COVID-19 *Bifidobacterium*, promotes efficacy of vaccines against SARS-CoV-2 (76). These results provided possible evidence for using probiotics for the defense of COVID-19. However, using probiotics for therapy is worth noting in many aspects. Although probiotics have an excellent overall safety record, they should be used with caution in certain patient groups like neonates born with immunodeficiency. Because of the paucity of information regarding the mechanisms through which probiotics act, appropriate administrative regimens, and probiotic interactions, further investigation is needed in these areas. Finally, note that the properties of different probiotic species vary and can be strain-specific. Therefore, careful consideration should be given to these issues before patients are advised to use probiotic supplements in clinical practice (77).

**Figure 3:**
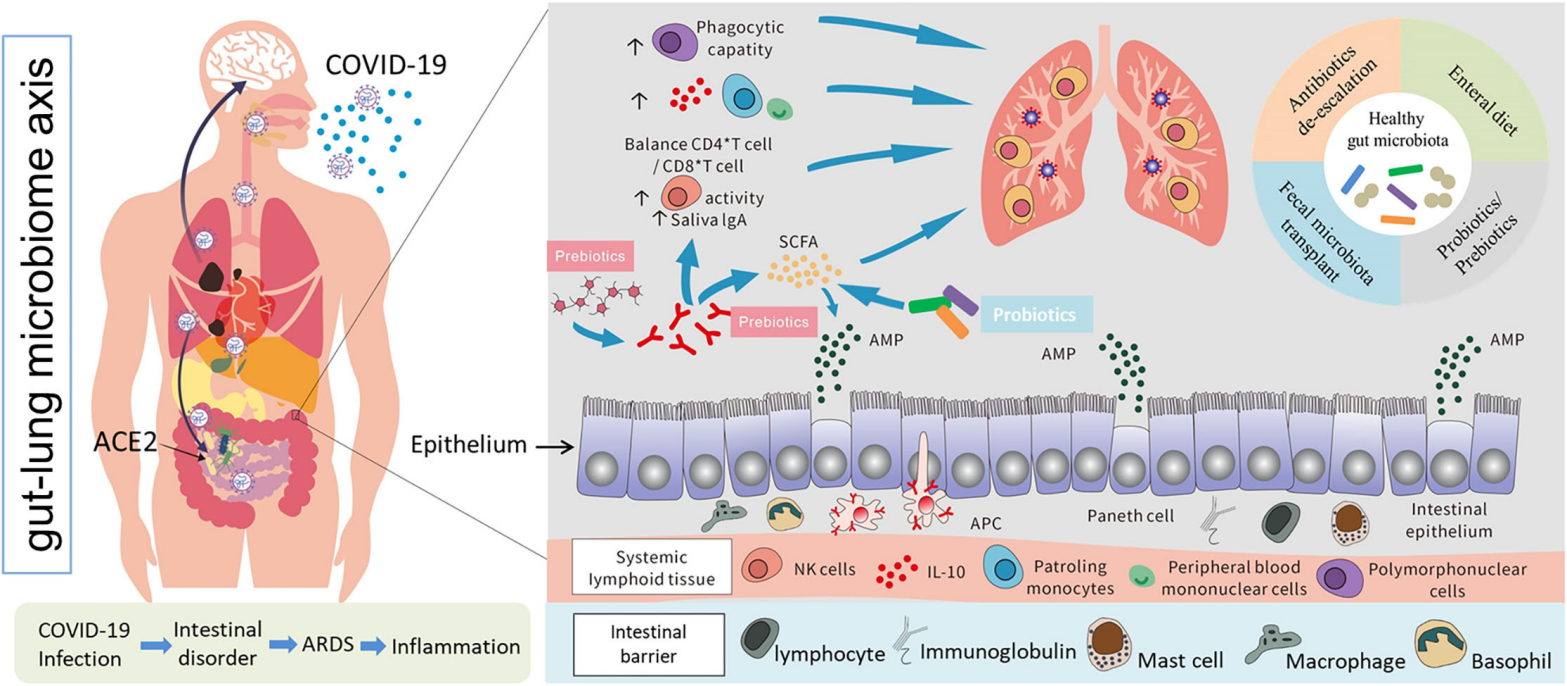
Possible models of SARS-CoV-2 infection, interactions between the human gut and lungs, and potential positive immune responses triggered by probiotics and other prebiotics against lung infection and injury (69, 70, 78–80).

The intestinal immune system is regulated by microbes and their metabolites. Anaerobic colonic bacteria are adopted to ferment non-digestible carbohydrates, such as cellulose, xylans, resistant starch, and inulin, to yield energy for microbial development (78). The most frequently used probiotics, such as lactic acid bacteria and bifidobacteria, can also generate SCFAs by fermenting carbohydrate end-products (79). The generated SCFAs, such as butyric acid and propionic acid, are the most important metabolites with many microbials, which play a significant role for adjusting the intestinal mucosal immune block and keeping their common functions during respiratory tract infections (63). Moreover, SCFAs can strengthen the number and function of Tregs, thus weakening excessive inflammation and immune response in airway diseases (80). Different dietary carbohydrates not digested by the host, called “prebiotics”, can selectively stimulate the development and metabolic activity of probiotic bacteria, such as bifidobacteria and lactobacilli. In fact, through combining probiotics-prebiotics (called symbiotic), predominant bacteria and the generation of SCFAs of fecal microbiota can be shifted in a model system of the human colon (81). The generation of SCFAs by these bacteria is a promising fundamental regulatory factor of epithelial proliferation in the gut (82). Except for SCFAs, a lot of other metabolites of the symbiotic gut microbiota associate with host immunity (83). Retinoic acid maintains intestinal immune homeostasis, as IgA production can be promoted by B and Treg cell growth (84). Tryptophan is an energy source for *Lactobacillus* that can produce ligands for an aryl hydrocarbon receptor, which maintains the homeostasis of the epithelial block and intraepithelial lymphocytes (85). Niacin has been reported to promote anti-inflammatory properties of colonic macrophages and DCs through GPR109A signaling and enable them to induce Treg cells and IL-10-producing T cells (86). During respiratory tract infections, lipopolysaccharides (LPS) act as part of the intestinal mucosal immune block to keep their common functions (87). In addition, lactate and pyruvate can strengthen the immune response by attracting the dendrite protrusion of small intestinal mononuclear cells (88). LPS are part of the intestinal mucosal immune barrier and maintain normal functions during respiratory tract infections (87). The various probiotics and metabolites for pulmonary infectious disease treatment are summarized in Table 2. This evidence demonstrated that dietary fiber could play a significant function in adjusting the gut microbiota further to regulate the intestinal barrier for virus infection and injury. In pathogenic SARS-CoV-2 infections, a healthy gut microbiota also plays a significant role against lung infection and injury (Figure 3).

**Table 2:** The functional of different probiotics, microbial products and dietary products for host immunity enhancement to against pulmonary infectious disease.

**Characteristics**	**Main functions**	**References**
**Probiotics**		
*Lactobacillus gasseri*	Enhanced inflammatory signals Enhanced antiviral immune reaction	(74)
*Lactobacillus rhamnosus*	Enhanced vaccine immune efficacy Enhanced antiviral immune reaction	(89)
*Lactobacillus casei*	Enhanced phagocytic and killing activity of alveolar macrophages Increased levels of IgA, IFN-γ, and TNF-α	(90)
*Bifidobacterium*	Enhanced vaccine immune efficacy	(25)
**Microbial metabolites**		
Short-chain fatty acids (SCFAs) (Butyrate, Propionate)	Maintenance of mucosal barrier Enhanced antiviral immune reaction Anti-inflammatory effect	(63)
Retinoic acid	Increased IgA level Treg cell development	(84)
Niacin	Anti-inflammatory effect Increased activity of macrophages and dendritic cells Development of T regulatory cells and IL-10-producing T cells	(86)
LPS	Enhance the mucosal immune response provide improved resistance against infection	(87)
Desaminotyrosine	Increased IFN-1	(48)
**Dietary products**		
Carbohydrate polymers	Increased SCFAs	(91)
Prebiotics	Increased SCFAs	(92)

## Conclusion and Future Prospects

In conclusion, as the third wave of coronavirus epidemic of the 21st century, after SARS and Middle East respiratory syndrome, the COVID-19 pandemic shows a great global social and economic pressure. Given the close association between viral replication and gastrointestinal immunity, a healthy intestinal barrier strategy targeting and modulating the host immune response may effectively decrease viral replication and its spread to the circulatory system. Additionally, probiotic uses have been reported as a promising strategy for severe COVID-19 infection treatment by China's National Health Commission and studies by the National Administration of Traditional Chinese Medicine. Probiotics improve the composition of the gut microbiota and the function of microbial metabolites. Most importantly, coronavirus-targeting vaccines and antiviral drugs have been developed to prevent COVID-19 and future epidemics (Table 3). Notably, according to several results, intestinal dysbiosis may affect the failure to respond to vaccines. With regard to this, the gut microbiota could influence intestinal immune responses as immune modulators and natural vaccine adjuvants. Therefore, the probiotic bacterial polysaccharide structure can be regarded as a lipopeptide-based vaccine. Thus, in the control of COVID-19 and other coronavirus-mediated diseases, it is potential for probiotic bacteria to strengthen vaccine efficacy.

**Table 3:** Clinical trials about using probiotics to regulate gut microbiota for COVID-19 treatment and COVID-19 vaccination efficacy.

**Identifier**	**Country/location**	**Intervention**	**Study design**	**Main aim**
NCT04366089	Italy	Probiotic	Parallel assignment	Treatment COVID-19
NCT04366180	Spain	Probiotic	Parallel assignment	Treatment COVID-19
NCT04399252	Unit States	Probiotic	Parallel assignment	Treatment COVID-19
NCT04420676	Austria	Synbiotic	Parallel assignment	Treatment COVID-19
NCT04980560	Prince of Wales Hospital, Hong Kong	Probiotic	An observation study	Compare microbiome profile in subjects with different COVID-19 vaccination and subjects recovered from COVID-19
NCT04884776	Prince of Wales Hospital, Hong Kong	3Bifidobacteria at 2 × 1,010 CFU for 12 weeks	Parallel assignment	Restore gut microbiota to increase COVID-19 vaccine efficacy and reduce side-effects
NCT04798677	Hospital Mare de Déu de la Merc, Spain	ABBC1 including beta-glucans, Inactivated saccharomyces cerevisae, Selenium, and Zinc	Parallel assignment	Enhance immune responses including generation of T cells, IgM and IgG

## Author Contributions

SZ and PF wrote the manuscript. WM revised the manuscript. XuL and WJ supervised the manuscript. XiL revised and supervised the manuscript. All authors approved the final manuscript.

## Funding

This work was supported by Fundamental Research Funds for the Central Universities, SCUT (No: lzujbky-2020-sp15), Gansu Province Major Science and Technology projects (No: 1602FKDA001), Science and Technology Project of Gansu Province (No: 18JR2TA018), Science and Technology Planning Project of Lanzhou Chengguan District (No: 2020JSCX0019) and Gansu Province CoVID-19 (NCP) Science and Technology Major Project (2020) (No: 20YF2FA008).

## Conflict of Interest

The authors declare that the research was conducted in the absence of any commercial or financial relationships that could be construed as a potential conflict of interest.

## Publisher's Note

All claims expressed in this article are solely those of the authors and do not necessarily represent those of their affiliated organizations, or those of the publisher, the editors and the reviewers. Any product that may be evaluated in this article, or claim that may be made by its manufacturer, is not guaranteed or endorsed by the publisher.
